# An Interannual Comparative Study on Ecosystem Carbon Exchange Characteristics in the Dinghushan Biosphere Reserve, a Dominant Subtropical Evergreen Forest Ecosystem

**DOI:** 10.3389/fpls.2021.715340

**Published:** 2021-10-18

**Authors:** Brian Njoroge, Yuelin Li, Shimin Wei, Ze Meng, Shizhong Liu, Qianmei Zhang, Xuli Tang, Deqiang Zhang, Juxiu Liu, Guowei Chu

**Affiliations:** ^1^Core Botanical Gardens, Chinese Academy of Sciences, Guangzhou, China; ^2^Key Laboratory of Vegetation Restoration and Management of Degraded Ecosystems, South China Botanical Garden, Chinese Academy of Sciences, Guangzhou, China; ^3^University of Chinese Academy of Sciences, Beijing, China

**Keywords:** Dinghushan, CO_2_ fluxes, Michaelis-Menten model, physiological process model, carbon sequestration

## Abstract

Compared with other forest systems, research interest in the potential for a stronger ecosystem carbon sequestration of evergreen forests throughout subtropical China has greatly increased. The eddy covariance technique is widely employed to determine accurate forest-atmosphere carbon dioxide (CO_2_) flux, which is subsequently used to determine forest ecosystem carbon exchange characteristics. The Dinghushan Biosphere Reserve, a subtropical monsoon evergreen broad-leaved forest, is a suitable study area due to its warm and humid climate (compared with other regions within the same latitude), consequently playing a role in the carbon cycle in the region. For this study, we hypothesized that the forest land in this region generally acts as a carbon sink, and that its carbon sequestration capacity increases over time despite the influence of climatic factors. Here, we compared net CO_2_ flux data derived from the eddy covariance technique over an 8-year study window. Additionally, we ascertained the effects of various environmental factors on net CO_2_ flux, while also using the Michaelis–Menten model and a physiologically based process model to track and report on ecosystem carbon exchange characteristics. We observed seasonal trends in daily ecosystem flux, indicative of sensitivity to climatic factors, such as air temperature, precipitation, and sunlight. The carbon sequestration capacity of the region exhibited seasonal variability, increasing from October to March (−264 g C m^−2^ year^−1^, i.e., 48.4%) while weakening from April to September (−150 g C m^−2^ year^−1^, i.e., 40.4%) on average. The net ecosystem exchange (NEE) rate varied from −518 to −211 g C m^−2^ year^−1^; ecosystem respiration (Re) varied from 1,142 to 899 g C m^−2^ year^−1^; and gross primary production (GPP) varied from 1,552 to 1,254 g C m^−2^ year^−1^. This study found that even though the Dinghushan Biosphere Reserve generally acts as a carbon sink, its carbon sequestration capacity did not increase significantly throughout the study period. The techniques (models) used in this study are suitable for application in other ecosystems globally, which can aid in their management and conservation. Finally, the Dinghushan Biosphere Reserve is both an exemplary and a model forest system useful in exploring CO_2_ absorption and sequestration from the atmosphere.

## Introduction

Recent research published by the scientific community on climate change has caused a general sense of alarm. Anthropogenic-derived greenhouse gases, such as carbon dioxide (CO_2_), are driving global climate change through contamination and alterations in atmospheric gaseous composition (Herndon, 2018; Kweku et al., 2018). Forest systems around the world, through their natural photosynthetic and respiratory activities, play a critical role in the extraction and sequestration of greenhouse gasses from the atmosphere (Domke et al., 2020; Moomaw et al., 2020). Research in China has determined that forest systems on the whole function as a carbon sink with an annual net C sequestration rate of 84 to 154 Tg C/year (e.g., 1 Tg = 10^12^ g) (Lu et al., 2018).

Tropical forest systems, such as the Dinghushan Biosphere Reserve in China, can be employed to tackle climate change, whose alterations can transform the entire planet into one which acts to endanger the whole biosphere (Fuss et al., 2021). Through means of the eddy covariance method as well as other flux measurement techniques, scientists can accurately quantify the CO_2_ that tropical forests sequester from the atmosphere (Li et al., 2008a, 2012; Kirschbaum et al., 2020). Given that well-preserved ecosystems, such as the Dinghushan Biosphere Reserve, are particularly sensitive to climate change, ecologists, in recent years, have become increasingly interested in investigating them (Malhi et al., 2020). The Dinghushan Biosphere Reserve, a subtropical monsoon evergreen forest, is unique because of its warmer climate compared with that of other regions of same latitude, consequently playing a significant role in the carbon cycle in the region (Li et al., 2012). Additionally, given that the Dinghushan Biosphere Reserve is a United Nations Educational, Scientific, and Cultural Organization (UNESCO) world heritage site that has been preserved for hundreds of years, it also acts as a uniquely rich research station (Li et al., 2012; Yang et al., 2021).

The balance between respiratory and assimilated carbon processes determines the net ecosystem exchange (NEE) rate of CO_2_, and this correlates to the complex interrelationships among the various factors, such as non-photosynthetic and photosynthetic plant tissues, plant species, meteorological conditions, CO_2_ exchange capacity, canopy structure, and plant biomass (Wilson et al., 2001; Baldocchi et al., 2002; Miao et al., 2010). Therefore, understanding the physical and biological controlling factors behind respiration and photosynthesis is critical to accurately estimate how environmental changes will affect NEE (Miao et al., 2010).

Owing to its comparative ease of use and low cost, the chamber method, used to measure ecosystem CO_2_ flux, has gained popularity in recent years; however, it is limited by its inherent small sample size restriction and the extensive labor that is necessary to collect enough data to represent an entire ecosystem (Zha et al., 2007; Tang et al., 2008; Miao et al., 2010). Another limitation of this method is that it requires many representative samples to obtain accurate mean estimates for ecosystem respiration while further being subject to chamber effects that lead to uncertainties, including environmental disturbances, pressure gradient alterations, and the introduction of turbulent fluctuations (Zha et al., 2007; Savage et al., 2008; Miao et al., 2010). For these reasons, this study used the eddy covariance technique.

The eddy covariance technique has been proven to be an accurate and effective method compared with its chamber-based counterpart in measuring respiration and ecosystem net CO_2_ exchange (Baldocchi et al., 2002; Wu et al., 2006; Miao et al., 2010). FLUXNET, an independent, global, and regional eddy covariance network, was established in 2002 and has since been applied to the Chinese Terrestrial Ecosystem Flux Research Network (ChinaFLUX), with which this study is associated, that uses eddy covariance data to estimate ecosystem CO_2_ flux as well as the utilization of its continuous flux information to estimate vertical turbulent CO_2_ flux between the atmosphere and the biosphere on an ecosystem scale (Baldocchi, 2003; Miao et al., 2010). Initially, CO_2_ flux measurements in the Dinghushan Biosphere Reserve started in 2002 and continues to this day (Li et al., 2012). It has, however, been found that eddy covariance accuracy is influenced by random errors associated with instrumentation, surface conditions, and atmospheric conditions, which have been factored into our analysis (Baldocchi et al., 1996; Zha et al., 2007). In addition to a standardized CO_2_ flux data collection system, Li et al. (2012) reported that a standardized data processing procedure is also needed for effective data comparisons among different biomes in China and from around the world.

The carbon uptake capacity of an ecosystem is defined by its gross primary production (GPP) and its ecosystem respiration (Re), whose roles in modeling are key to the extrapolation of carbon dynamics and the understanding of ecosystems holistically (Gilmanov et al., 2003). Data, such as NEE, leaf area index (LAI), and maximum electron transport rate during photosynthesis (Vc_max_), can be included in these models to estimate ecosystem carbon exchange and the potential of an ecosystem to adapt to such changes, and to simulate present and future ecosystem carbon balances (Owen et al., 2007).

In this study, we hypothesize that the Dinghushan Biosphere Reserve generally acts as a carbon sink and that its carbon sequestration capacity has increased over time despite the influence of climatic factors. The objectives of this study were as follows: (1) to compare net interannual CO_2_ flux data derived from the eddy covariance technique over an 8-year study period (2003–2010), (2) to ascertain the effects of various environmental factors on net CO_2_ flux, and (3) to utilize the Michaelis–Menten model (i.e., the hyperbolic light response model) and a physiologically based process model to track and report on ecosystem carbon exchange characteristics.

## Materials and Methods

### Site Description

For this study, a flux tower was constructed in a mixed coniferous-broadleaved forest located within the Dinghushan Biosphere Reserve. The area of the region is 1,156 ha, located between latitudes 23°09′21″ and 23°11′30″N and longitudes 112°30′39″ and 112°33′41″E, Guangdong province, within the southern subtropical region of China. The climate is humid with a maximum temperature of 38°C, a minimum temperature of −0.2°C, and an annual average temperature of 20.9°C (Li et al., 2012). Maximum precipitation and warm temperatures span from April to September (the rainy season), and minimum precipitation and cold temperatures span from October to March (the dry season). The mean annual precipitation of the region is 1,927 mm (Tang et al., 2006). The elevation of the region is between 100 and 700 m and its topography primarily comprises hills and mountains. Soil pH ranges from 4.5 to 6, and the soil type is primarily lateritic, consisting of a humus layer. The coniferous and broad-leaved forest investigated for this study is 400 years old. Its average canopy height is 17 m, and the maximum LAI ranges from 3.8 to 4.2 m^−2^m^−2^ at a 95% canopy density (Li et al., 2012). The dominant tree species are *Castanopsis chinensis, Pinus massoniana, Schima superba, Cryptocarya concinna*, and *Machilus chinensis*; moreover, the flora is rich, consisting of >1,740 plant species (Zou et al., 2018).

### CO_2_ Flux Measurements Using the Eddy Covariance Method

Between 2003 and 2010, this study systematically measured latent heat, CO_2_ exchange, and energy using the open-path eddy covariance system following the procedures outlined by Li et al. (2008b). Two sensors were used: a CSAT3 (Campbell Scientific, Inc., North Logan, UT, USA) sonic anemometer and a LI-7500 (LI-COR Inc., Lincoln, NE, USA) open-path CO_2_ analyzer mounted on an 80 × 80-cm mast placed at a height of 38 m. At a precise frequency of 10 Hz, water vapor concentration, air temperature, and CO_2_ were measured and recorded. Quality tests and planar fit rotations were performed using the updated methodology reported by Foken and Wichura (1996). At 30-min intervals, CO_2_ concentration and vertical velocity covariances were calculated, and a data-screening routine was applied to exclude periods that showed weak turbulence (u^*^), namely, lower than 0.2 m s^−1^. Under the guidelines established by Aubinet et al. (1999), Lee et al. (2004), and Mauder and Foken (2006), the EUROFLUX methodology was used to calculate NEE applying flux data. A negative NEE value was indicative of carbon gains from the atmosphere to the ecosystem, suggesting that the ecosystem acted as a carbon sink. A gap-filling procedure was conducted by employing the marginal distribution sampling method. Additionally, using a short-term temperature-dependent method, GPP and Re were computed from NEE (Reichstein, 2001; Owen et al., 2007). Flux data were recorded as either g C m^−2^ or μmol CO_2_ m^−2^ s^−1^ per unit time.

### Meteorological Data Collection

All meteorological data were collected from five belowground soil layers and at heights of 4, 9, 15, 21, 27, 31, and 36 m above the soil surface (Baldocchi et al., 1988). A CM11 (Kipp & Zonen, Sterling, VA, USA) pyranometer and a CNR1 (Kipp & Zonen, Sterling, VA, USA) net radiometer were placed on top of the tower wherein solar radiation was measured. Precipitation was also measured from the top of the tower using a tipping bucket rain gauge (52203, R.M. Young Company, Traverse City, MI, USA). LQS70-10 (Apogee, Logan, UT, USA) and Li190SB (LI-COR Inc., Lincoln, NE, USA) sensors were used to measure photosynthetically active radiation (PAR). Wind velocity was measured using an A100R (Vector Instruments, UK) cup anemometer, while wind direction was measured using a W200P wind vane (Vector Instruments, UK). The HMP45C (Campbell Scientific Inc., North Logan, UT, USA), and IRTS-P (Apogee, Logan, UT, USA) sensors were used to measure temperature and humidity at the aforementioned levels. The 105-T and 107-L (Campbell Scientific Inc., North Logan, UT, USA) and the CS616 (Campbell Inc., USA) probes were used to measure soil temperature and soil moisture, respectively, at a depth of 5, 20, 40, 80, and 100 cm. The CR10X (3) and CR23X (1) data loggers (Campbell Scientific Inc., North Logan, UT, USA) were used to record meteorological signals and data averaged into 30-min average values.

### The Michaelis–Menten Model (Rectangular Hyperbola)

The Michaelis–Menten model is also referred to as the hyperbolic light response model, and it is useful in describing measured, un-gap filled NEE flux obtained *via* the eddy covariance method, particularly that measured during daytime hours, by comparing flux to the radiation received by fitting it into a non-linear least-square fitted model that provides empirical parameters as described in the equation shown in Table 1 (Gilmanov et al., 2003; Owen et al., 2007; Li et al., 2008a).


$$\begin{array}{l} \text{NEE}=\frac{\alpha \beta Ǫ}{\text{α}Ǫ+\beta }+\gamma \end{array}$$

To avoid any interference from gap-filling routines, only un-gap-filled NEE data were used in our analysis. For context, gap-filled flux is best used to visualize trends over various periods and was, therefore, utilized in this specific analysis and elsewhere where applicable. Estimations of parameters α, β, and γ were mainly conducted using high-quality daytime NEE flux data during both the dry season and the rainy season in the Dinghushan Biosphere Reserve to compare seasonal effects on these parameters. During the dry (winter) season, if the parameters exceeded 0.17 for α, 100 for β, and 15 for γ, or if the parameters had a standard error >0.6, they would be rejected, because they were assumed unsuitable to fit the rectangular hyperbola model as recommended by previous studies (Owen et al., 2007; Li et al., 2008b).

**Table 1:** Parameters obtained from the Michael–Menten model and their meaning and units.

**Parameter**	**Meaning**	**Unit**
NEE	Net ecosystem exchange	μmol CO_2_ m^−2^ s^−1^
α	The initial slope of the light response curve and approximation of light utilization efficiency	μmol CO_2_/μmol photon m^−2^ s^−1^
β	The maximum CO_2_ uptake rate of the canopy	μmol CO_2_ m^−2^ s^−1^
γ	Estimate of average daytime ecosystem respiration	μmol m^−2^s^−1^
O̧	Photosynthetic photon flux density (PPFD)	μmol photon m^−2^ s^−1^
(β + γ)_2000_	Average maximum canopy uptake capacity (GPP)	μmol CO_2_ m^−2^ s^−1^
(α + β)	Radiation required for the half-maximal uptake rate	–
(β + γ)	Theoretical maximum uptake capacity	μmol CO_2_ m^−2^ s^−1^

### Physiologically Based Process Model

This model is single-layered, describing leaf gas exchanges and light interception by defining the sunlit and shaded classes of given canopy foliage. The model was fitted using inputs, such as global radiation, soil temperature, air temperature, wind speed, relative humidity, atmospheric CO_2_ level, air pressure, LAI, and GPP (short-term gross uptake flux was obtained by summing Re and NEE) measured at the site using the following equation (Owen et al., 2007; Li et al., 2008a,b):


$$\begin{array}{l} {\text{F}}_{\text{GPP}}={\text{F}}_{\text{NEE}}+{\text{F}}_{\text{Re}} \end{array}$$

Because the Dinghushan Biosphere Reserve is located within a subtropical climate, its corresponding LAI values do not fluctuate significantly from season to season and are measured to be between 3.8 and 4.2 m^−2^m^−2^. Using the absorption and emission of long-wave radiation, the interception of light, convective heat loss, and heat loss through transpiration, the energy balance of leaves was calculated into two classes, namely, sunlit and shaded (Chen et al., 1999; Owen et al., 2007). Gross photosynthesis was simulated by the procedures described by Farquhar and Caemmerer (1982) and refined further for application in the field by Harley and Tenhunen (1991) using the following equation:


$$\begin{array}{l} P_{m}=\frac{alpha\;I}{\sqrt{1+\left(\frac{alpha^{2}I^{2}}{P_{ml}^{2}}\right)}} \end{array}$$

where *I* is the incident photosynthetic photon flux density (PPFD); *alpha* is the mean leaf light use efficiency (LUE); *P*_*ml*_ is the light and CO_2_ dependent potential of the RuBP regeneration rate as clarified by Falge (1997); and *P*_*m*_ is the gross photosynthesis. Using enzyme reactions of Ribulose-1,5-bisphosphate-carboxylase-oxygenase (Rubisco) as a basis for the parameters used for model inversions, Reichstein (2001) and Owen et al. (2007) had explicated the nuances of the model where the rate of CO_2_ fixation is constrained by either a high internal CO_2_ concentration or the regeneration of RuBP at low light intensity. Next, using values derived by Owen et al. (2007) in their leaf gas exchange experiments under conditions devoid of water limitations, the response of leaf and stomatal temperatures were held constant. A model inversion routine was performed using *alpha, Vc*_*uptake*_, and site *F*_*GPP*_ data, which were pooled into the years investigated and subsequently into the particular season being investigated (rainy season or dry season). To eliminate noise in parameter estimation originating from prevailing weather conditions during measurement periods, we adopted the guidance provided by Owen et al. (2007) and Li et al. (2008a), reducing the number of parameters in the physiological model to one where *alpha* was dependent on *Vc*_*uptake*_. A linear relationship between *alpha* and *Vc*_*uptake*_ was then established from parameter estimations as follows:


$$\begin{array}{l} \alpha =Vc_{uptake}\;0.0006 \end{array}$$

Using this equation, a single parameter (*Vc*_*uptake*_1^*^) obtained from model inversion, which was consistent with Owen et al. (2007), was calculated for each year investigated and for each season in the Dinghushan Biosphere Reserve.

### Statistical Analysis

Linear and non-linear regression, the hyperbolic light response model (i.e., the Michaelis–Menten model), and all statistical analyses were conducted using SigmaPlot® for Windows version 14.0 build 14.0.0.124. The physiologically based process model was generated using the PV WAVE® analysis software (32-bit version) with code written on a matching IDL® console that shares a common programming syntax. Standard and polynomial quadratic curve functions were used for all regression analyses. For ease of visualization, the negative exponential smoother with a sampling proportion of 0.1 and a polynomial degree of 1 was used to smooth daily CO_2_ flux (NEE, GPP, and Re).

## Results

### Climate

**Figure 1:**
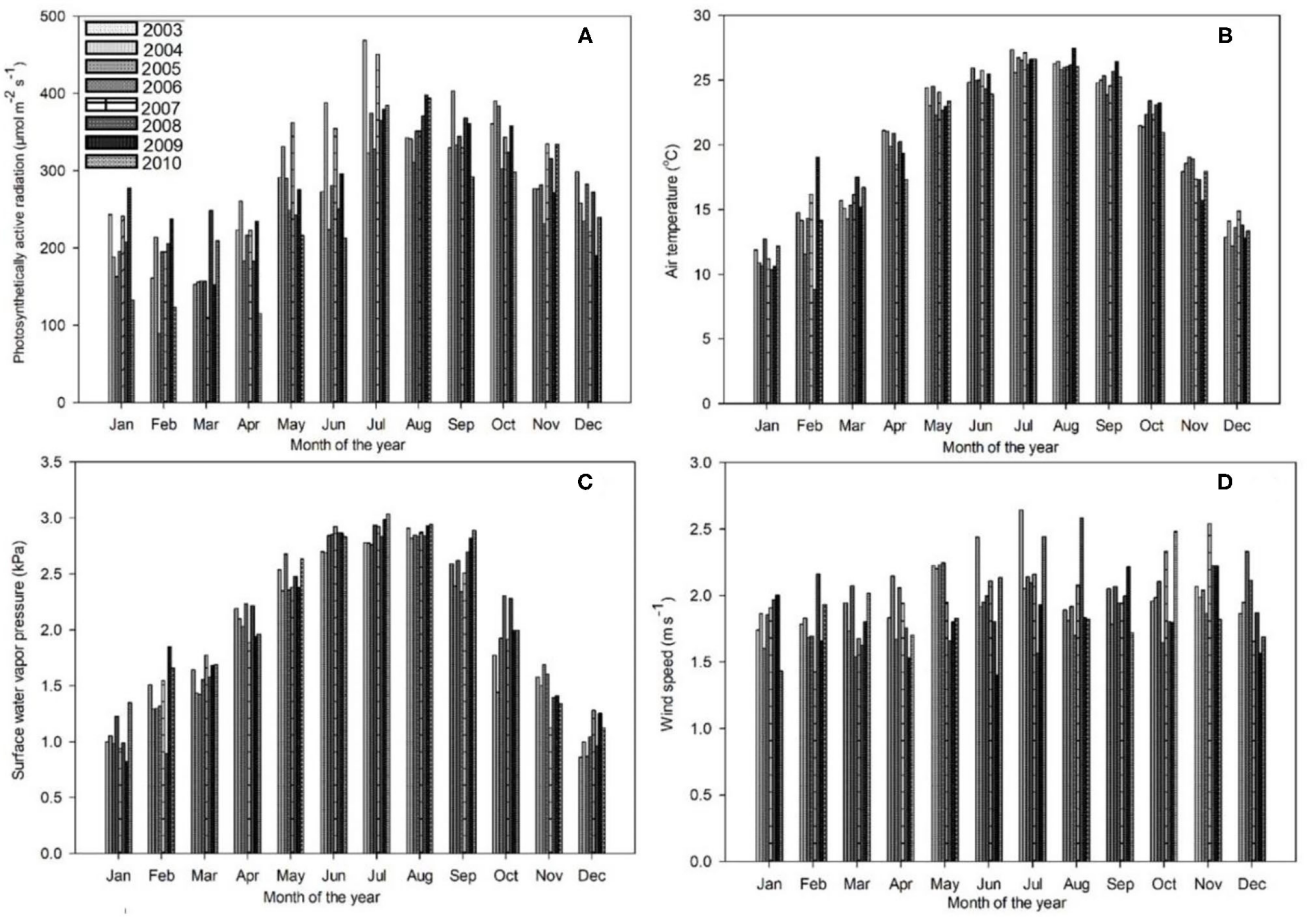
Trends in monthly daytime mean values of **(A)** photosynthetically active radiation (PAR) (μmol m^−2^s^−1^), **(B)** air temperature (°C), **(C)** surface water vapor pressure deficit (VPD) (kPa), and **(D)** wind speed (ms^−1^) for the Dinghushan Biosphere Reserve at an aboveground height of 38 m, measured between 2003 and 2010.

**Figure 2:**
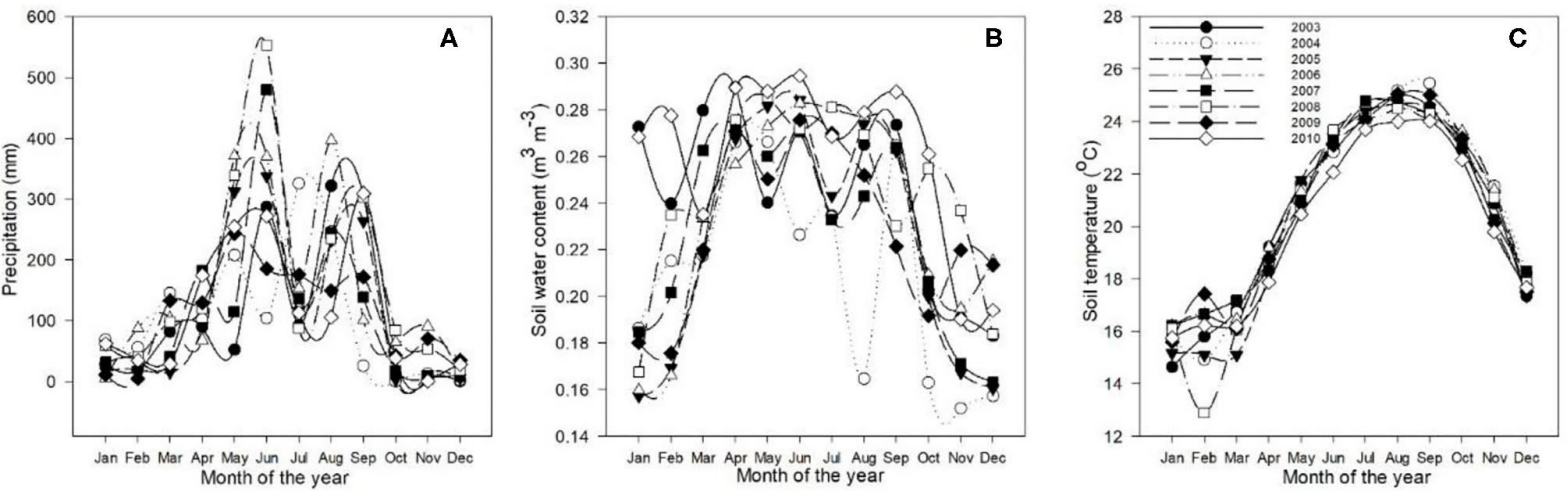
Annual trends in **(A)** precipitation (mm), **(B)** soil water content (m^3^m^−3^), and **(C)** soil temperature (°C) at a 5-cm depth for the Dinghushan Biosphere Reserve from 2003 to 2010.

Figures 1, 2 reveal the climatic conditions in the Dinghushan Biosphere Reserve from 2003 to 2010. PAR, air temperature, surface water vapor pressure, and wind speed all exhibited a similar trend for all the years investigated. Moreover, PAR was highest in July 2003 (Figure 1A), whereas air temperature peaked in August 2009 (Figure 1B). The highest surface vapor pressure was recorded in July 2010 (Figure 1C), while the fastest wind speed was measured in July 2003 (Figure 1D). Precipitation, soil water content, and soil temperature all exhibited a similar trend between 2003 and 2010. The highest precipitation was recorded in July 2008 (Figure 2A), the highest soil water content was recorded in June 2010 (Figure 2B), and the highest soil temperature was recorded in September 2004 (Figure 2C).

Figure 2A shows two clear annual precipitation peaks, one in May and one in September, which provides the time of year with the highest precipitation for all the 8 years investigated. The highest soil temperatures (Figure 2C) were measured in August and September. As a general observation, between 2008 and 2010, the annual seasonal climate patterns between April and September were hot and humid and between October to March were cool and dry. Seasonal temperature variation was between 26.6°C during hot and humid months and 11.3°C during cool and dry months, when large storms were observed during hot and humid months. The maximum wind speed was 2.09 ms^−1^, while solar radiation and the vapor pressure deficit varied between 168 and 384.4 μmol m^−2^ s^−1^ and between 1.05 and 2.88 kPa, respectively.

### Measured CO_2_ Flux

**Figure 3:**
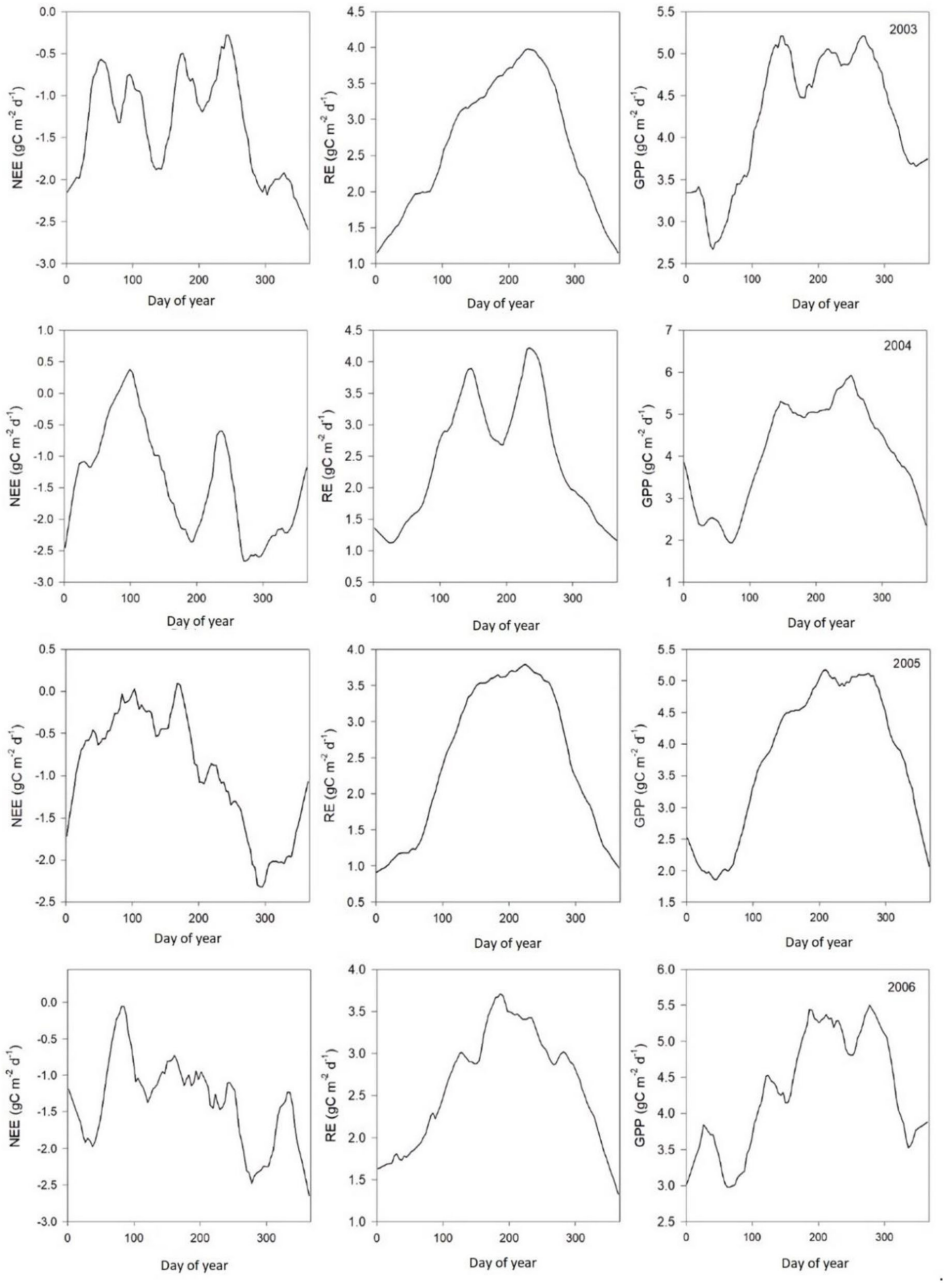
Average daily ecosystem carbon dioxide (CO_2_) flux patterns (μmol m^−2^s^−1^) between 2003 and 2006.

**Figure 4:**
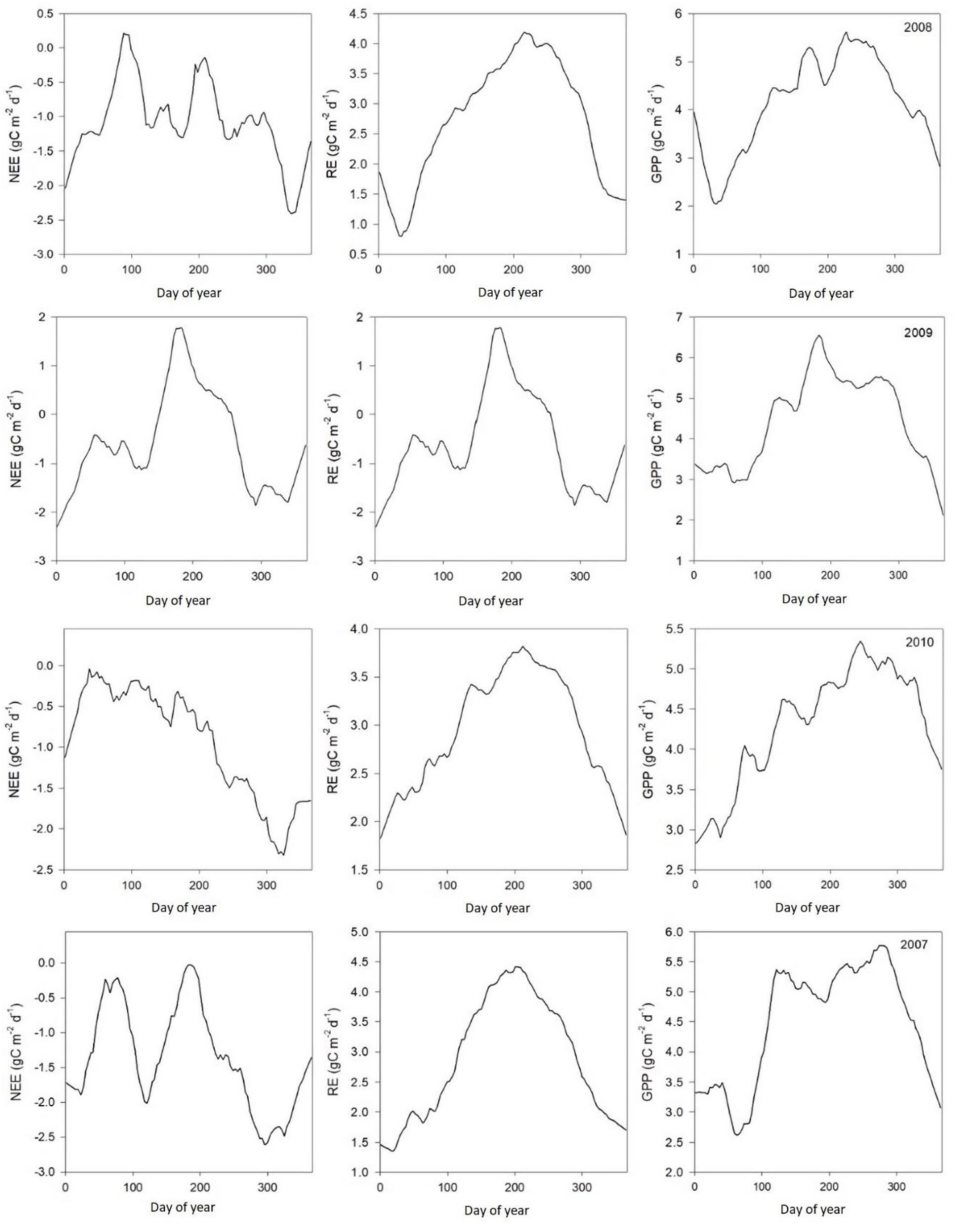
Average daily ecosystem CO_2_ flux patterns (μmol m^−2^s^−1^) between 2007 and 2010.

Table 2 shows monthly mean NEE, Re, and GPP values from 2003 to 2010. Results from Figures 3, 4 show clear seasonal trends in daily ecosystem flux, which indicate sensitivity to climatic factors between 2003 and 2004. The results showed that the Dinghushan Biosphere Reserve was a net CO_2_ sink throughout the year, with a monthly average of 119.84g C m^−2^ month^−1^ and an annual sequestration average of −413.2 g C m^−2^ year^−1^. The carbon sequestration capacity of the region varied seasonally, namely, from October to March (cool and dry months), thus exhibiting a greater carbon sink capacity of −263.55 g C m^−2^ year^−1^ (48.4%), whereas warmer and wetter months (from April to September) had a weaker overall carbon sink capacity of −149.65 g C m^−2^ year^−1^ (40.4%).

**Table 2:** Monthly average carbon dioxide (CO_2_) flux; units used for net ecosystem exchange (NEE), ecosystem respiration (Re), and gross primary production (GPP) are expressed in μmol m^−2^s^−1^; SE are shown in parentheses; negative NEE values indicate that the Dinghushan Biosphere Reserve is a carbon sink.

	**2003**			**2004**			**2005**			**2006**			**2007**			**2008**			**2009**			**2010**		
	**NEE**	**RE**	**GPP**	**NEE**	**RE**	**GPP**	**NEE**	**RE**	**GPP**	**NEE**	**RE**	**GPP**	**NEE**	**RE**	**GPP**	**NEE**	**RE**	**GPP**	**NEE**	**RE**	**GPP**	**NEE**	**RE**	**GPP**
Jan	−1.96 (0.22)	1.37 (0.05)	3.39 (0.19)	−1.45 (0.22)	1.17 (0.04)	2.76 (0.19)	−0.92 (0.17)	1.01 (0.03)	2.14 (0.12)	−1.56 (0.21)	1.67 (0.10)	3.42 (0.18)	−1.85 (0.22)	1.33 (0.07)	3.33 (0.19)	−1.51 (0.14)	1.27 (0.11)	2.81 (0.18)	−1.71 (0.18)	1.23 (0.09)	2.97 (0.21)	−0.72 (0.16)	2.11 (0.07)	2.99 (0.15)
Feb	−0.61 (0.19)	1.75 (0.08)	2.63 (0.14)	−1.11 (0.15)	1.42 (0.04)	2.57 (0.13)	−0.61 (0.13)	1.18 (0.05)	1.86 (0.13)	−1.71 (0.19)	1.80 (0.07)	3.54 (0.21)	−0.88 (0.24)	2.04 (0.07)	3.33 (0.15)	−1.28 (0.12)	1.01 (0.09)	2.29 (0.14)	−0.91 (0.21)	2.54 (0.09)	3.72 (0.15)	−0.15 (0.18)	2.30 (0.14)	3.09 (0.19)
Mar	−1.22 (0.23)	1.97 (0.06)	3.37 (0.18)	−0.10 (0.03)	1.77 (0.06)	1.94 (0.06)	−0.27 (0.15)	1.56 (0.06)	2.31 (0.09)	−0.21 (0.21)	2.06 (0.11)	3.04 (0.14)	−0.17 (0.16)	2.07 (0.14)	2.81 (0.17)	−0.40 (0.21)	2.22 (0.08)	3.24 (0.13)	−0.62 (0.21)	1.98 (0.15)	2.89 (0.22)	−0.42 (0.30)	2.60 (0.10)	4.05 (0.19)
Apr	−1.01 (0.30)	2.66 (0.08)	4.24 (0.23)	0.29 (0.10)	2.93 (0.05)	3.46 (0.08)	−0.02 (0.22)	2.53 (0.06)	3.49 (0.11)	−1.22 (0.25)	2.66 (0.09)	4.01 (0.23)	−1.48 (0.27)	2.45 (0.14)	4.10 (0.31)	−0.28 (0.31)	2.74 (0.11)	4.028 (0.25)	−0.71 (0.25)	2.75 (0.12)	4.10 (0.19)	−0.01 (0.23)	2.72 (0.08)	3.69 (0.18)
May	−1.75 (0.22)	3.14 (0.05)	4.97 (0.21)	−0.93 (0.23)	3.72 (0.14)	4.96 (0.17)	−0.64 (0.17)	3.28 (0.06)	4.23 (0.13)	−1.04 (0.24)	2.93 (0.06)	4.32 (0.18)	−1.54 (0.27)	3.56 (0.09)	5.40 (0.22)	−0.87 (0.21)	3.12 (0.09)	4.32 (0.18)	−0.93 (0.26)	3.57 (0.09)	4.97 (0.17)	−0.53 (0.24)	3.46 (0.05)	4.62 (0.16)
Jun	−0.67 (0.27)	3.33 (0.05)	4.57 (0.19)	1.99 (0.15)	3.07 (0.09)	5.09 (0.15)	0.16 (0.23)	3.52 (0.04)	4.53 (0.14)	−0.78 (0.26)	3.43 (0.12)	4.72 (0.21)	−0.58 (0.19)	4.15 (0.12)	5.13 (0.14)	−1.31 (0.32)	3.54 (0.08)	5.36 (0.28)	1.10 (0.22)	4.37 (0.13)	5.65 (0.28)	−0.27 (0.23)	3.32 (0.03)	4.32 (0.13)
Jul	−1.07 (0.15)	3.73 (0.06)	4.86 (0.13)	−2.36 (0.08)	2.68 (0.04)	5.04 (0.07)	−0.88 (0.24)	3.65 (0.06)	4.99 (0.15)	−1.09 (0.32)	3.51 (0.08)	5.32 (0.18)	−0.20 (0.10)	4.34 (0.06)	4.84 (0.06)	−0.23 (0.15)	3.94 (0.13)	4.54 (0.18)	1.16 (0.23)	4.62 (0.09)	5.93 (0.02)	−0.80 (0.17)	3.75 (0.06)	4.82 (0.10)
Aug	−0.70 (0.17)	3.98 (0.03)	4.94 (0.12)	−0.79 (0.27)	4.16 (0.09)	5.53 (0.19)	1.03 (0.15)	3.78 (0.05)	4.91 (0.14)	−1.42 (0.28)	3.40 (0.05)	5.27 (0.17)	−1.52 (0.19)	4.07 (0.11)	5.60 (0.21)	−1.05 (0.24)	4.02 (0.08)	5.57 (0.16)	0.46 (0.14)	4.87 (0.10)	5.48 (0.09)	−1.25 (0.17)	3.69 (0.05)	5.01 (0.13)
Sep	−0.76 (0.27)	3.71 (0.03)	5.11 (0.15)	−1.88 (0.28)	3.42 (0.14)	5.62 (0.14)	1.28 (0.20)	3.54 (0.04)	5.08 (0.14)	−1.63 (0.29)	3.02 (0.08)	4.99 (0.21)	−1.46 (0.26)	3.63 (0.06)	5.43 (0.17)	−1.19 (0.18)	3.88 (0.09)	5.30 (0.14)	−0.03 (0.20)	4.54 (0.08)	5.34 (0.12)	−1.22 (0.20)	3.60 (0.07)	5.06 (0.15)
Oct	−2.11 (0.19)	2.71 (0.09)	4.92 (0.14)	−2.63 (0.10)	2.09 (0.05)	4.72 (0.11)	−2.36 (0.12)	2.55 (0.10)	4.91 (0.14)	−2.36 (0.22)	2.92 (0.06)	5.34 (0.18)	−2.50 (0.21)	3.03 (0.10)	5.61 (0.18)	−1.13 (0.21)	3.25 (0.06)	4.74 (0.12)	−1.88 (0.18)	3.42 (0.06)	5.41 (0.13)	−1.90 (0.21)	3.17 (0.09)	5.20 (0.16)
Nov	−1.94 (0.18)	2.08 (0.06)	4.10 (0.14)	−2.20 (0.13)	1.77 (0.03)	3.98 (0.12)	−2.03 (0.14)	1.89 (0.06)	3.94 (0.12)	−1.36 (0.26)	2.41 (0.07)	4.04 (0.21)	−2.42 (0.12)	2.14 (0.07)	4.56 (0.12)	−1.61 (0.24)	2.02 (0.13)	3.88 (0.14)	−1.51 (0.18)	2.14 (0.19)	3.77 (0.17)	−2.26 (0.11)	2.57 (0.04)	4.83 (0.10)
Dec	−2.28 (0.13)	1.37 (0.03)	3.65 (0.12)	−1.84 (0.12)	1.28 (0.04)	3.12 (0.12)	−1.56 (0.14)	1.17 (0.04)	2.74 (0.15)	−2.11 (0.17)	1.68 (0.06)	3.83 (0.15)	−1.74 (0.18)	1.83 (0.07)	3.60 (0.15)	−2.05 (0.20)	1.47 (0.06)	3.55 (0.20)	−1.37 (0.21)	1.54 (0.08)	3.10 (0.18)	−1.66 (0.24)	2.20 (0.08)	4.04 (0.21)

**Figure 5:**
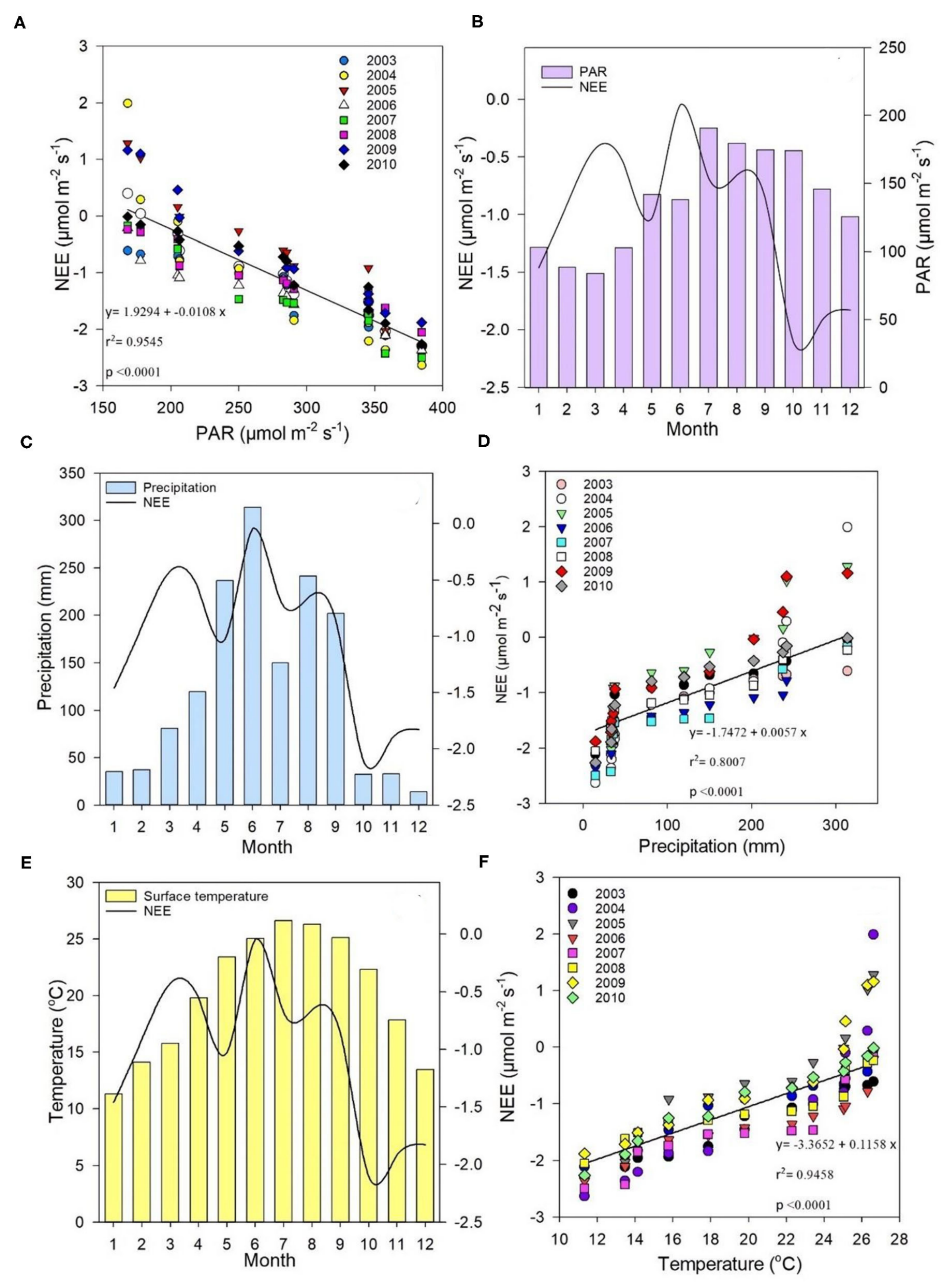
**(A)** Correlation between measured monthly daytime net ecosystem exchange (NEE) (μmol m^−2^s^−1^) and monthly average midday PAR (μmol m^−2^s^−1^); **(B)** trends in average daily NEE (μmol m^−2^s^−1^) compared with average monthly PAR (μmol m^−2^s^−1^); **(C)** relationship between monthly average precipitation (mm) and daily average NEE (μmol m^−2^s^−1^); **(D)** correlation between NEE (μmol m^−2^s^−1^) and precipitation (mm); **(E)** relationship between monthly temperature (°C) and daily NEE averages (μmol m^−2^s^−1^); **(F)** correlation between NEE (μmol m^−2^s^−1^) and temperature (°C) between 2003 and 2010.

A linear correlation between measured monthly daytime NEE and monthly average midday PAR is shown in Figure 5A, wherein averages were calculated when the potential maximum photosynthetic capacity was between 1,100 to 1,300 of the land surface temperature (LST). PAR ranged from 168.01 to 384.4 μmol m^−2^s^−1^, and NEE ranged from 0.4 to −2.29 μmol m^−2^s^−1^, with a linear relationship of *r*^2^ = 0.9545 at *p* < 0.0001. Figure 5B provides the daily NEE trend in comparison with average monthly PAR, illustrating that an increase in PAR during warmer months would increase NEE. However, there were other climatic factors that clearly affected CO_2_ flux (i.e., not PAR alone). By overlaying the NEE trend, monthly precipitation patterns could be subdivided into two (Figures 2A, 5C), which showed that precipitation strongly affected ecosystem CO_2_ flux. Regression analysis also showed that precipitation impacted NEE [*r*^2^ = 0.8007 at *p* < 0.0001 (Figure 5D)]. The correlation between temperature and NEE indicated that temperature also impacted ecosystem CO_2_ flux [*r*^2^ = 0.9458 at *p* < 0.0001 (Figure 5F)]. On the other hand, Figure 5E shows that although temperature impacted NEE significantly, it did not fully account for all NEE fluctuations, demonstrating that climate factors collectively had a net combined effect on NEE, namely, lower precipitation and temperatures contributed to lower NEE.

**Figure 6:**
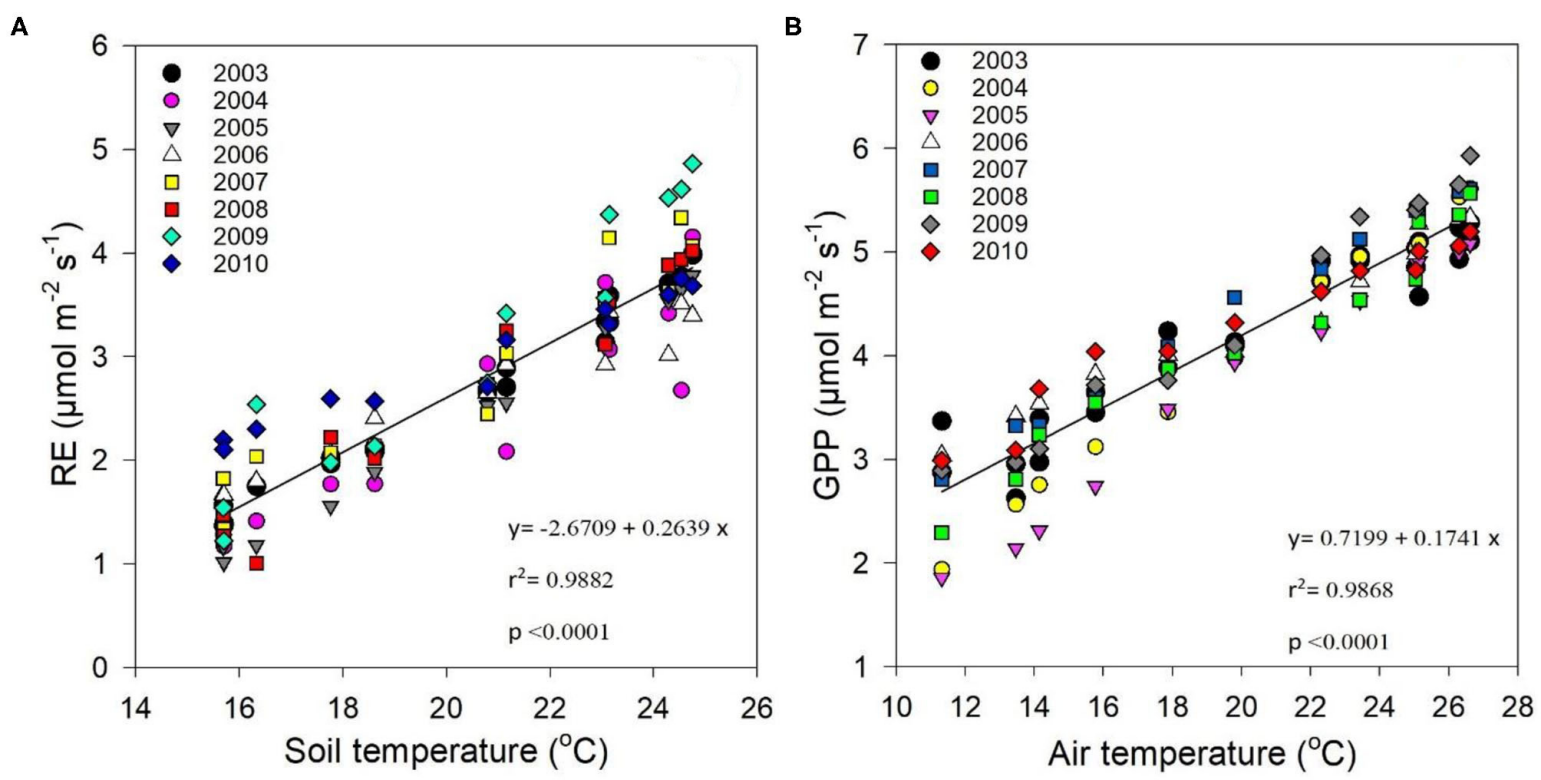
Correlation between **(A)** average daily ecosystem respiration (Re) (μmol m^−2^s^−1^) and monthly soil temperature (°C), and **(B)** average daily GPP (μmol m^−2^s^−1^) and monthly air temperature (°C) between 2003 and 2010.

Regression analysis between Re and soil temperature at a soil depth of 5 cm exhibited an exponential relationship [*r*^2^ = 0.9882 at *p* > 0.0001 (Figure 6A)], showing that soil temperature significantly impacted Re. On the other hand, Figure 6B shows test results for the relationship between GPP and air temperature, and demonstrates that seasonal temperature variations impacted GPP (*r*^2^ = 0.9868 at *p* > 0.0001).

### The Michaelis–Menten and Physiologically Based Models

This study revealed that monsoon evergreen forests, such as the Dinghushan Biosphere Reserve, can be characterized by multiple variables that correlate in a myriad of complex ways. This study used the Michaelis–Menten model to simplify relationship variability.

**Figure 7:**
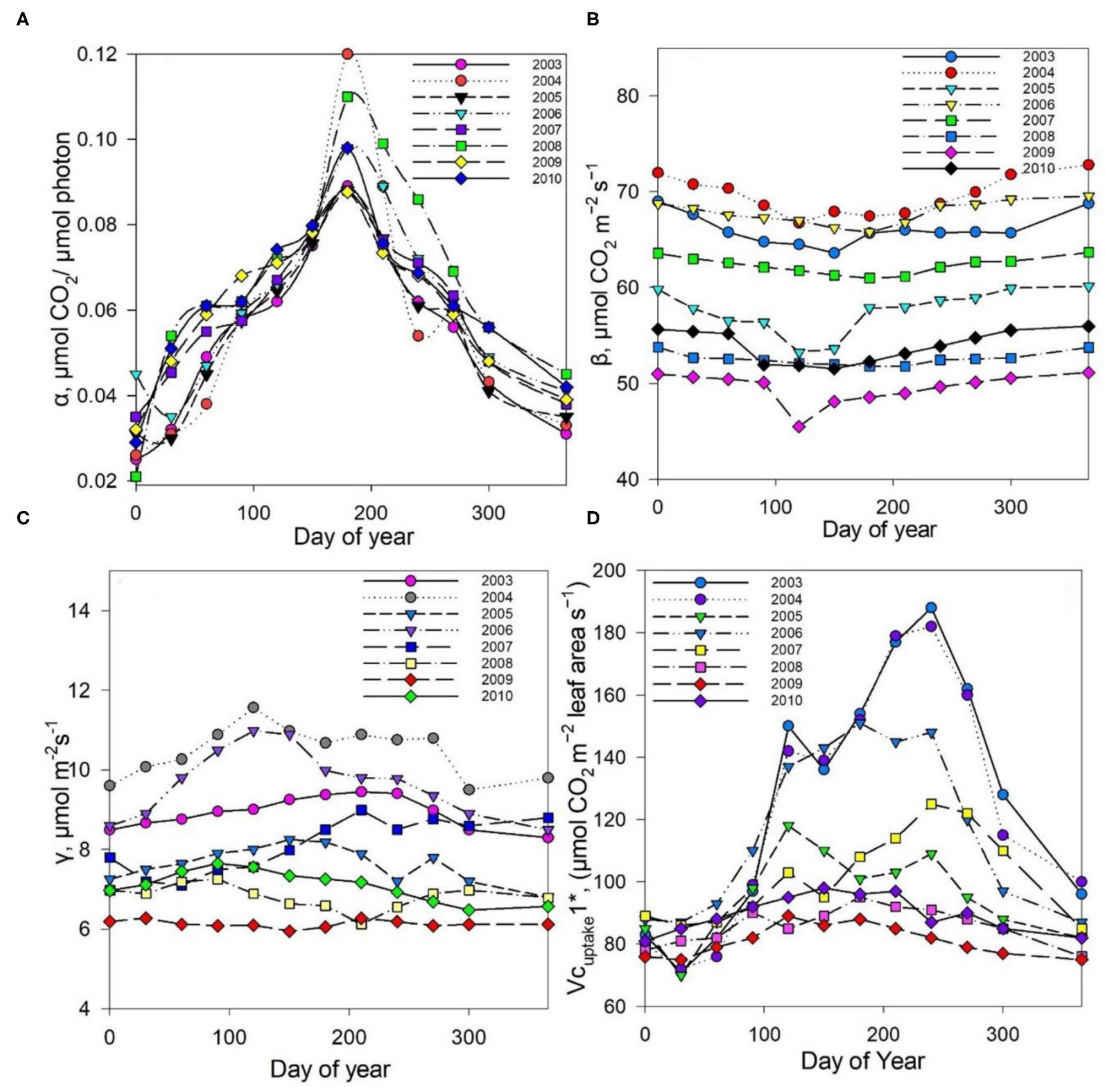
Seasonal trends in parameters **(A)** α alpha, **(B)** β beta, **(C)** γ gamma, and **(D)** *Vc*_*uptake*_1* throughout the study period (2003–2010).

**Figure 8:**
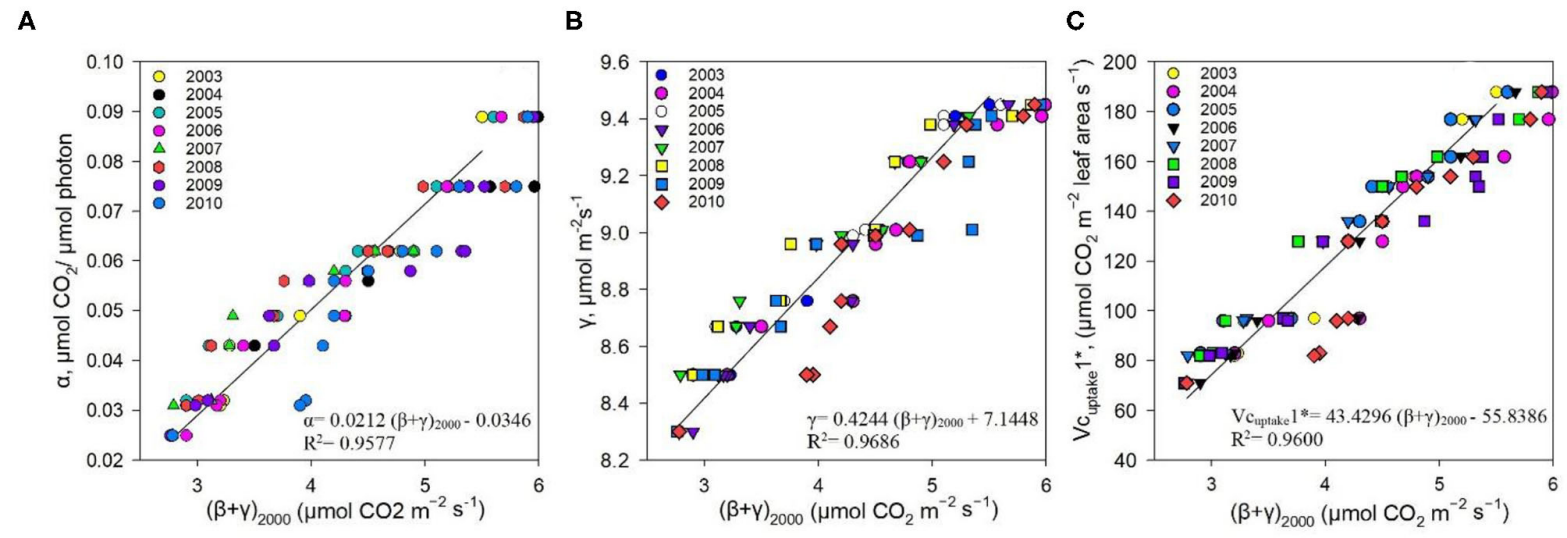
Correlation between **(A)** α and (β + γ)_2000_, **(B)** γ and (β + γ)_2000_, and **(C)** *Vc*_*uptake*_1* and (β + γ)_2000_ between 2003 and 2010.

**Figure 9:**
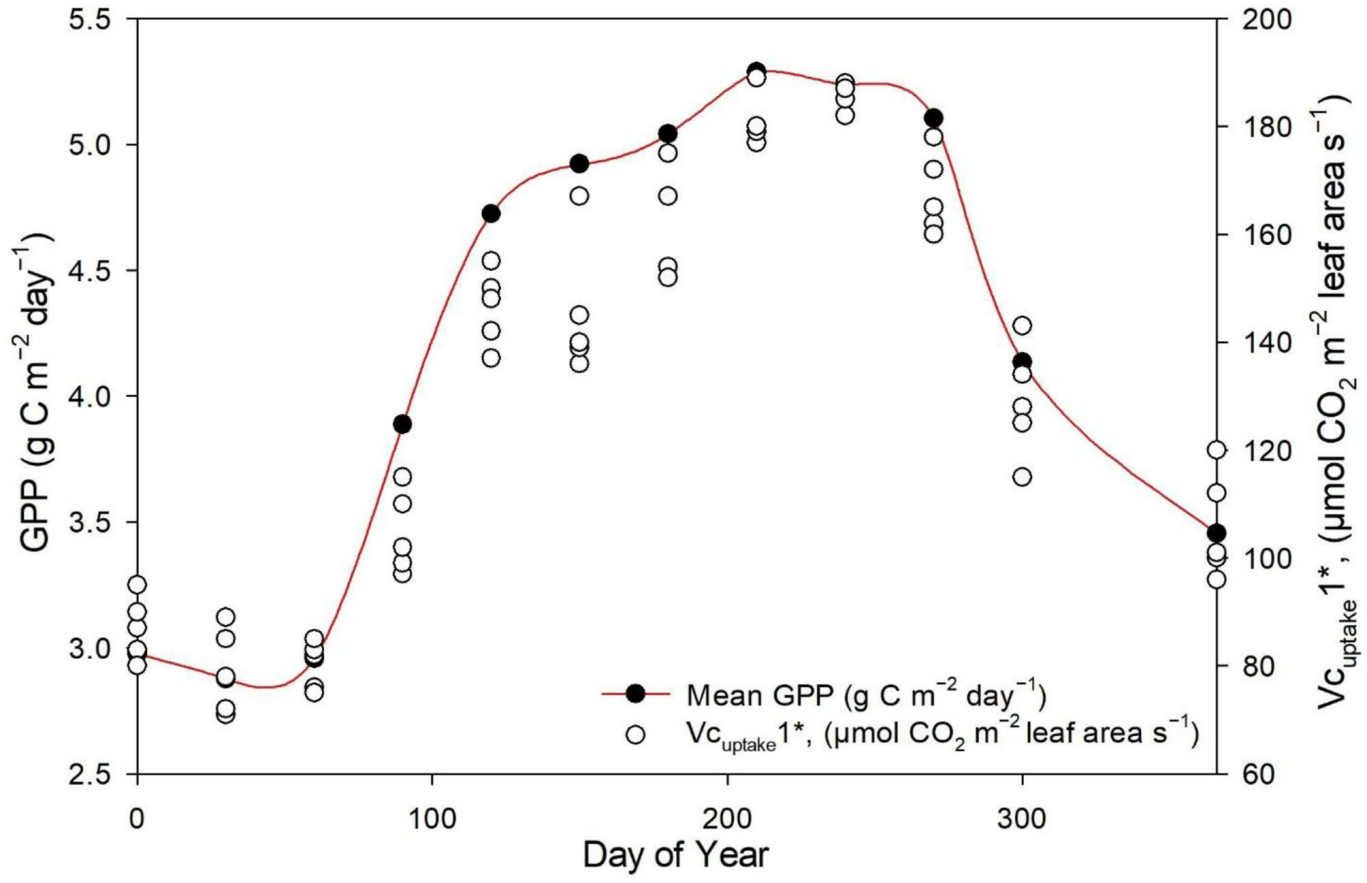
Seasonal trends in measured GPP (μmol m^−2^s^−1^) and *Vc*_*uptake*_1* between 2003 and 2010.

The measured NEE varied from −518.57 to −211.57 g C m^−2^ year^−1^; measured Re varied from 1,142.83 to 899.98 g C m^−2^ year^−1^; and GPP varied from 1,552.94 to 1,254 g C m^−2^ year^−1^ over the 8-year study period (Table 3). The measurements showed that the Dinghushan Biosphere Reserve is a strong carbon sink. In Figure 7A, α was highest during the warmer and wetter months of June, July, and August and decreased significantly during the drier and colder months of December, January, and February, while following a similar trend throughout the study period. In Figure 7B, β followed a comparable trend but was significantly higher in 2004, 2006, and 2003 compared with the other years investigated, wherein its lowest value was observed in 2009. As shown in Figure 7C, seasonal γ (gamma) trends exhibited slight fluctuations as the seasons transitioned from rainy to dry, where the highest γ value was observed in 2004 and the lowest γ was in 2009. *Vc*_*uptake*_1^*^ peaked in 2003 and 2004, and slightly lagged as a reaction to this peak during the rainy season (Figure 7D), while the lowest *Vc*_*uptake*_1^*^ value was observed in 2009, namely, when compared to the other years investigated. Figures 8A–C show that α, β, and *Vc*_*uptake*_1^*^ strongly correlated to (β + γ)_2000_, with *r*^2^ values of 0.9577, 0.9686, and 0.96, respectively, and that this correlation remained strong throughout the study period. This means that a change in (β + γ)_2000_ (defined in Table 1) will have a strong effect on α, β, and *Vc*_*uptake*_1^*^. Seasonal trends in measured GPP through ecosystem flux and *Vc*_*uptake*_1^*^ followed a similar pattern throughout the 8-year study period, peaking during the warm and rainy seasons (approximately June, July, and August) and decreasing during the colder and drier months (Figure 9).

**Table 3:** Measured and modeled NEE (g C m^−2^ y^−1^), Re (g C m^−2^ y^−1^), and GPP (g C m^−2^ y^−1^) from 2003 to 2010.

**Year**	**Measured NEE**	**Modeled NEE**	**NEE R^**2**^**	**Measured GPP**	**Modeled GPP**	**GPP R^**2**^**	**Measured RE**	**Modeled RE**	**RE R^**2**^**
2003	−491.71	−467.71	0.95	1,460.63	1,416.63	0.95	968.92	948.92	0.85
2004	−518.57	−496.57	0.99	1,418.54	1,373.54	0.89	899.97	876.97	0.99
2005	−349.41	−328.41	0.85	1,254.00	1,211.00	0.85	904.59	882.59	0.89
2006	−501.14	−478.14	0.99	1,460.02	1,417.02	0.99	958.88	938.88	0.96
2007	−497.58	−477.58	0.85	1,552.94	1,514.94	0.85	1,055.36	1,037.36	0.85
2008	−393.63	−374.63	0.99	1,387.86	1,409.99	0.95	994.22	1,035.36	0.99
2009	−211.56	−192.56	0.87	1,354.39	1,231.92	0.87	1,142.83	1,039.36	0.87
2010	−342.01	−323.01	0.99	1,422.73	1,298.23	0.99	1,080.72	975.22	0.95
Average	−413.20	−392.33	0.94	1,413.89	1,359.16	0.92	1,000.69	966.83	0.92

## Discussion

### Relationship Between CO_2_ Flux and Meteorological Conditions

Results from the gap-filling methods showed that meteorological conditions impacted ecosystem carbon flux (Figures 3, 4), with optimum temperature, precipitation, and sunlight (PAR) having the greatest overall influence on NEE in the Dinghushan Biosphere Reserve. Seasonal weather pattern variations and changes in overall climate conditions, such as increase in annual temperatures and shift in precipitation patterns, have been shown to impact ecosystems in Belgium and North America, which is also evident in subtropical ecosystems of South China (Aubinet et al., 2002; Gough et al., 2008). The maximum PAR recorded at the research station occurred between 11 a.m. and 1 p.m., with an average maximum of 384.41 μmol m^−2^s^−1^ and a concurrent average maximum NEE of −2.29 μmol m^−2^s^−1^, where both parameters exhibited a strong linear correlation throughout the 8-year study period (Figure 5A). Studies conducted in shrubland and evergreen forests at various latitudes support our findings in addition to showing that PAR had an almost immediate effect on NEE while influencing carbon sequestration on a short-term scale (Ouyang et al., 2014; Jia et al., 2018). On the other hand, the temperature has been shown to cause NEE to lag, namely, taking ~2–3 h for the air temperature to effect NEE on a daily scale (Ouyang et al., 2014) and an average of 19 days on an annual scale (Jia et al., 2018). In the Dinghushan Biosphere Reserve, temperature had an impact on ecosystem CO_2_ flux (*r*^2^ = 0.9458 at *p* < 0.0001; Figure 5F). Ecosystem NEE values, such as those of grassland in semiarid regions, have been found to be highly sensitive to precipitation and soil moisture content under an increase in precipitation conditions, causing an increase in NEE (Oquist et al., 2014; Fang et al., 2018). However, Oquist et al. (2014) reported that increased precipitation occurs alongside increased cloud cover, which significantly affects PAR and therefore leads to a reduction in NEE. This reduction in PAR due to cloud cover does not seem to be highly significant in the Dinghushan Biosphere Reserve (according to the findings shown in Figure 5B), and this is perhaps the result of increased transpiration and stomatal conductance during the rainy season that influences gas exchanges and, hence, NEE. Figures 3, 4 show that the Dinghushan Biosphere Reserve is a strong CO_2_ sink, which is a curious and unique phenomenon, during the cold and dry seasons,. Studies on this unique phenomenon have not been conducted in the Dinghushan Biosphere Reserve; however, Doughty and Goulden (2008) offer interesting insight that in part helps to explain our observations. Their study reported that dryer and colder months of the year caused ‘leaf flushing' to occur, where older leaves fell and exposed younger leaves lower in the canopy, which resulted in higher levels of gaseous exchange that subsequently resulted in an increase in NEE. Studies conducted on seasonal changes in the albedo effect and near infrared (NIR) reflectance of forest systems provides an explanation as to why there is an observed increase in carbon uptake during dry and cold seasons (i.e., winter) (Tanaka et al., 2003; Doughty and Goulden, 2008). Forests influenced by Asian monsoons during dry and cold seasons are comparatively darker in the visible spectrum but brighter with respect to NIR, while some forests actually absorb more PAR during such seasons, thus appearing darker and more photosynthetically active, hence, producing higher NEE measurements (Tanaka et al., 2003).

### Michaelis–Menten and Physiologically Based Models

**Figure 10:**
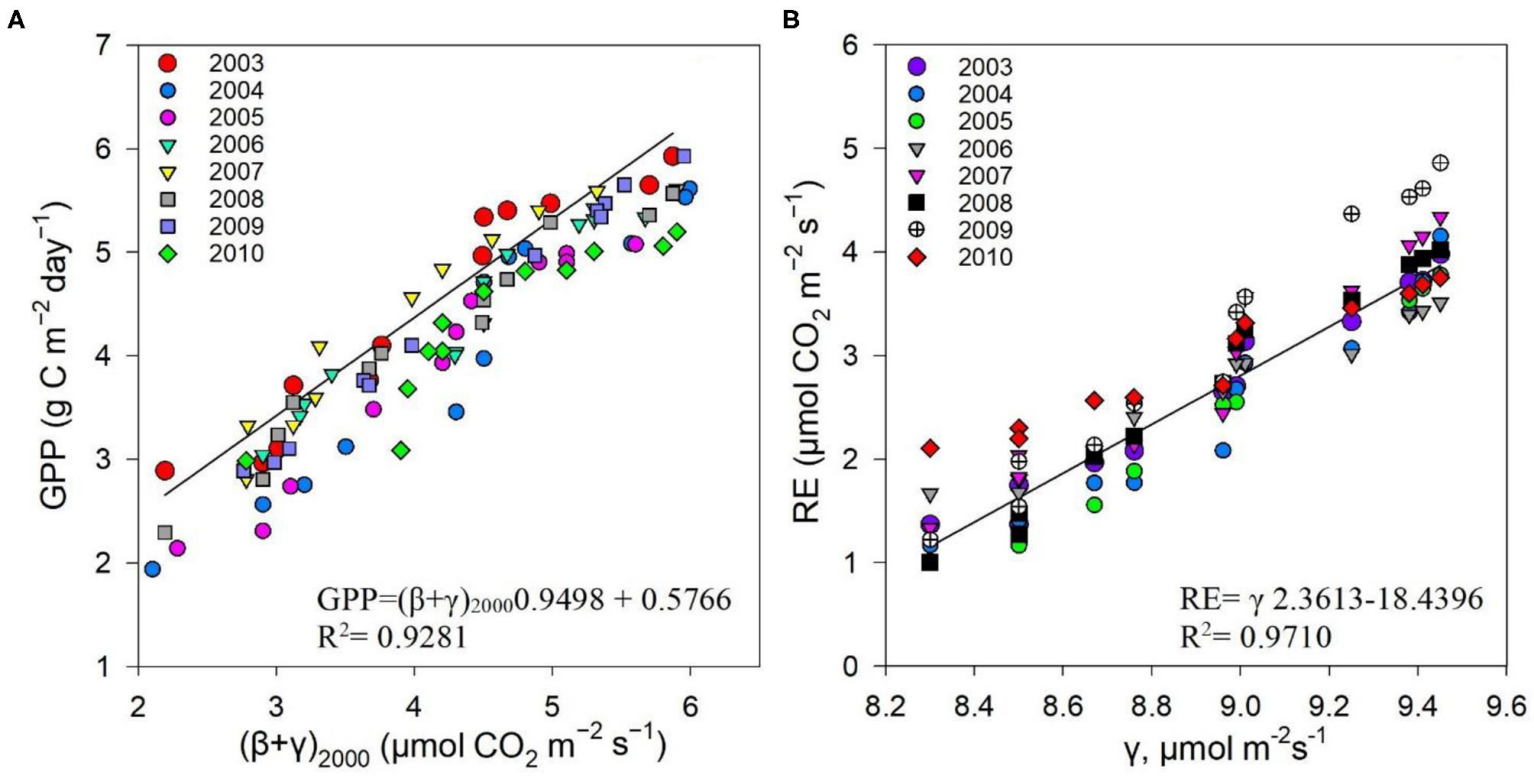
Correlations between **(A)** measured GPP (μmol m^−2^s^−1^) and (β + γ)_2000_, **(B)** measured Re (μmol m^−2^s^−1^), and γ between 2003 and 2010.

The non-rectangular hyperbola model containing an additional curvature parameter (η) has been shown in previous studies to provide more representative α, β, and γ values (Gilmanov et al., 2003; Owen et al., 2007). However, the rectangular hyperbola was specifically chosen for this study to simplify the model for application in a subtropical evergreen forest ecosystem, namely, the Dinghushan Biosphere Reserve, using the value η = 0. The measured GPP showed a strong correlation to (β + γ)_2000_ (Table 1, Figure 10A), and the measured Re showed a strong correlation to γ (Figure 10B), leading to the conclusion that the hyperbolic light response model (i.e., the Michaelis–Menten model), as confirmed by other studies, is well-suited for exploring ecosystem CO_2_ characteristics over short-term and long-term studies (Gilmanov et al., 2003; Li et al., 2008a).

## Conclusions

The Dinghushan Biosphere Reserve is a net carbon sink; however, its carbon sequestration capacity has neither increased nor shown any discernable trend throughout this 8-year analysis. Instead, 2004, 2006, and 2007 were observed to have the highest annual NEE values of −518.57, −501.14, and −497.58 g C m^−2^ a^−1^, respectively (Table 2, Figures 3, 4). This forest system has been shown to be a strong CO_2_ sink during cold and dry seasons (i.e., winter) (Table 4) (Figures 3, 4), making it an interesting case study and necessitating more research to be done to clarify the exact mechanisms that underlie this unique and counterintuitive phenomenon. Furthermore, further studies on carbon characteristics at a timescale of 20 years or more are necessary for the Dinghushan Biosphere Reserve to establish the impact that climate change has had on carbon uptake and sequestration, and to document what this means for other tropical forest ecosystems. The models used in this study are sufficiently suitable for studying other ecosystems around the world to help in their management, conservation, and sustainable policy-making decisions *via* relevant authorities (Figures 7–9). In the race against climate change, the Dinghushan Biosphere Reserve will prove to be a great tool as well as a model forest system for the absorption and sequestration of CO_2_ from the atmosphere.

**Table 4:** Parameter values of the hyperbolic light response model (i.e., the Michaelis–Menten model) and the physiologically based process model, from 2003 to 2010.

	**2003**		**2004**		**2005**		**2006**		**2007**		**2008**		**2009**		**2010**	
	**Wet Season**	**Dry season**	**Wet Season**	**Dry season**	**Wet Season**	**Dry season**	**Wet Season**	**Dry season**	**Wet Season**	**Dry season**	**Wet Season**	**Dry season**	**Wet Season**	**Dry season**	**Wet Season**	**Dry season**
*n*	34	35	42	32	28	39	45	39	34	39	41	34	47	48	41	49
α	0.089	0.069	0.11	0.098	0.073	0.063	0.099	0.086	0.078	0.065	0.076	0.062	0.069	0.059	0.074	0.064
β	63.98	65.69	69.99	72.32	57.79	59.68	65.12	68.87	59.99	62.72	50.86	52.56	48.02	50.37	51.98	55.89
*β_2, 000_*	32.11	35.66	30.47	36.56	22.98	26.89	32.91	36.54	28.63	30.95	18.96	20.73	22.92	24.81	18.72	21.34
(β + γ)_2000_	4.35	4.11	4.08	3.97	3.89	3.45	4.39	4.12	4.59	4.67	4.38	4.65	4.12	4.46	4.59	4.28
γ	9.94	8.97	12.59	11.58	8.12	7.61	10.98	9.93	8.76	7.29	7.12	6.02	6.19	5.93	7.5	7.1
*R^2^*	0.89	0.85	0.97	0.99	0.87	0.85	0.79	0.84	0.93	0.91	0.94	0.89	0.82	0.84	0.97	0.94
VC_*uptake*_1*	138	131	182	179	109	103	151	145	125	121	95	89	76	71	98	91
LAI (m^−2^m^−2)^	4.2	3.8	4.1	3.9	4.2	3.8	4.1	3.8	4.2	3.9	4.1	3.8	4.2	3.9	4.1	3.8

## Data Availability Statement

The raw data supporting the conclusions of this article will be made available by the authors, without undue reservation.

## Author Contributions

BN and YL: data processing and writing. SW, QZ, and GC: data collection. ZM: instrument maintenance. SL: site maintenance. XT, DZ, and JL: experimental design. All authors contributed to the article and approved the submitted version.

## Funding

This study was funded by the Strategic Priority Research Program of the Chinese Academy of Sciences, Grant No. XDA23080302; the National Science and Technology Basic Work Project, Grant No. 2015FY1103002; the National Natural Science Foundation of China (31961143023, 31670453, and 41430529); the Dinghushan Forest Ecosystem Positioning Research Station of the National Science and Technology Infrastructure Platform, the Chinese Ecosystem Research Network (CERN); and the Operation Service Project of the National Scientific Observation and Research Field Station of the Dinghushan Forest Ecosystem of Guangdong, the Ministry of Science and Technology of the People's Republic of China.

## Conflict of Interest

The authors declare that the research was conducted in the absence of any commercial or financial relationships that could be construed as a potential conflict of interest.

## Publisher's Note

All claims expressed in this article are solely those of the authors and do not necessarily represent those of their affiliated organizations, or those of the publisher, the editors and the reviewers. Any product that may be evaluated in this article, or claim that may be made by its manufacturer, is not guaranteed or endorsed by the publisher.
